# When Are Adverse Outcome Pathways and Associated Assays “Fit for Purpose” for Regulatory Decision‐Making and Management of Chemicals?

**DOI:** 10.1002/ieam.4153

**Published:** 2019-07-17

**Authors:** Katie Coady, Patience Browne, Michelle Embry, Thomas Hill, Eeva Leinala, Thomas Steeger, Lidka Maślankiewicz, Tom Hutchinson

**Affiliations:** ^1^ Toxicology & Environmental Research & Consulting Dow Chemical Company Midland Michigan USA; ^2^ Environment, Health and Safety Division, Environment Directorate Organisation for Economic and Cooperative Development Paris France; ^3^ Health and Environmental Sciences Institute Washington DC USA; ^4^ US Environmental Protection Agency National Health and Environmental Effects Research Laboratory, Research Triangle Park North Carolina; ^5^ Environment, Health and Safety Division, Environment Directorate Organisation for Economic and Cooperative Development Paris France; ^6^ US Environmental Protection Agency, Office of Pesticide Programs Washington DC; ^7^ National Institute of Public Health and the Environment (RIVM) Centre for Safety of Substances and Products, Bilthoven The Netherlands; ^8^ University of Plymouth Plymouth United Kingdom

**Keywords:** Adverse outcome pathway, Chemical decision making, Regulatory science, Fit for purpose, Defined approaches

## Abstract

There have been increasing demands for chemical hazard and risk assessments in recent years. Chemical companies have expanded internal product stewardship initiatives, and jurisdictions have increased the regulatory requirements for the manufacture and sale of chemicals. There has also been a shift in chemical toxicity evaluations within the same time frame, with new methodologies being developed to improve chemical safety assessments for both human health and the environment. With increased needs for chemical assessments coupled with more diverse data streams from new technologies, regulators and others tasked with chemical management activities are faced with increasing workloads and more diverse types of data to consider. The Adverse Outcome Pathway (AOP) framework can be applied in different scenarios to integrate data and guide chemical assessment and management activities. In this paper, scenarios of how AOPs can be used to guide chemical management decisions during research and development, chemical registration, and subsequent regulatory activities such as prioritization and risk assessment are considered. Furthermore, specific criteria (e.g., the type and level of AOP complexity, confidence in the AOP, as well as external review and assay validation) are proposed to examine whether AOPs and associated tools are fit for purpose when applied in different contexts. Certain toxicity pathways are recommended as priority areas for AOP research and development, and the continued use of AOPs and defined approaches in regulatory activities are recommended. Furthermore, a call for increased outreach, education, and enhanced use of AOP databases is proposed to increase their utility in chemicals management. *Integr Environ Assess Manag* 2019;15:633–647. © 2019 The Authors. *Integrated Environmental Assessment and Management* published by Wiley Periodicals, Inc. on behalf of Society of Environmental Toxicology & Chemistry (SETAC)

## Key Points

- Specific criteria are proposed for evaluating whether adverse outcome pathways (AOPs) and associated assays are fit for purpose in various chemical management scenarios.
- Case studies are provided to illustrate how AOPs may be applied in various stages of chemical management and decision making.
- In the future, priority should be given to developing AOPs that are clear and concise as well as relevant to regulatory endpoints and a large number of chemicals and associated modes of action.
- Adverse outcome pathways are useful in many different chemical management scenarios, and AOPs should continue to be used in regulatory efforts such as defined approaches.

## INTRODUCTION

With the growth of green chemistry initiatives (i.e., the design of chemical products and processes that reduce or eliminate the use or generation of hazardous substances) there is an increased demand for chemical hazard and risk assessments during early phases of chemical research and development (Carney and Settivari 2013). In addition, there are increased regulatory requirements for chemical hazard and risk assessments for new and existing chemicals (i.e., the Frank R Lautenberg Chemical Safety for the 21st Century Act in the United States; the Registration, Evaluation, Authorization and Restriction of Chemicals [REACH] in Europe; and REACH‐like regulations in parts of Europe and Asia Pacific [Sullivan et al. 2011; Brown et al. 2016]). This increased information demand is occurring at a time when there is also pressure to reduce, refine, and replace animal use in chemical toxicity testing (Sullivan et al. 2011; USEPA 2016a). These coinciding pressures have led to the development of alternative approaches (i.e., in vitro, in silico and in chemico methods) to assess chemicals with the aim to protect human and environmental health (Carney and Settivari 2013; Lillicrap et al. 2016). Gathering information from diverse data streams and drawing conclusions on potential hazard to guide chemical decision making and management can pose a challenge, especially for regulators faced with a large number of chemical assessments and chemical companies that share responsibility for ensuring chemical safety for humans and the environment.

**Figure 1:**
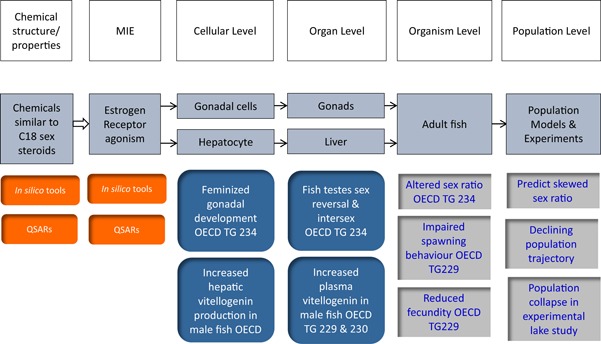
Estrogen receptor agonist adverse outcome pathway in fish with some examples of overlaid assays for assessment of key events and key event relationships (OECD 2009a, 2009b, 2011). MIE = molecular initiation event; OECD TG = Organisation for Economic Co‐operation and Development Test Guideline.

The Adverse Outcome Pathway (AOP) is a conceptual framework to systematically organize available data and knowledge that describes scientifically plausible relationships across multiple levels of biological organization between a molecular initiating event (MIE) and subsequent key events (KEs), culminating in an adverse outcome (AO) (Ankley et al. 2010). The AOP framework has been used to integrate data from different biological levels and guide chemical decision making. In addition, based on the increasing understanding of underlying biology and causal linkages between KEs, AOPs can inform the development and application of assays used to measure responses at various points along the pathway (Figure 1) and facilitate efforts to extrapolate across key events leading to an AO.

Although the AOP framework can be used to organize data and guide decisions such as selection of study endpoints or toxicity testing plans, there are still challenges in applying AOPs to chemical safety scenarios. In particular, applying AOPs to regulatory decisions remains challenging as evidenced by a debate at a Society of Toxicology meeting, (http://toxchange.toxicology.org/p/bl/et/blogid=9&blogaid=2569), which highlighted, among other things, the limited number of publicly available AOPs. Several previous publications have examined the potential applications and limitations of AOPs in chemical decision making (Patlewicz et al. 2015; Perkins et al. 2015; Edwards et al. 2016; Kleinstreuer et al. 2016). These publications reviewed the application of AOPs in regulatory contexts ranging from prioritization, categorization, application to integrated testing strategies, and quantitative risk assessment and highlighted the need for an increased stringency of validation. However, additional guidance is needed for determining “fit for purpose” for both AOPs and associated tools.

A SETAC Pellston Workshop® entitled “Advancing the Adverse Outcome Pathway Concept – An International Horizon Scanning Approach” (2–6 April 2017, Cornwall, Ontario) was held in order to advance the science and applications of AOPs (LaLone, Ankley et al. 2017), and the present paper summarizes workgroup discussions surrounding the application of AOPs in chemical management and regulatory decision making. Specifically, the purpose of the workgroup was to identify scenarios in which the AOP framework is useful in decision making and to propose criteria that can be used by chemical decision makers to identify whether an AOP is fit for specific decision contexts. The workgroup identified both the regulators (i.e., government agencies) and the regulated community (i.e., chemical industry) as key stakeholders and decision makers in a variety of chemical management activities, and potential recommendations for increasing the use and application of AOPs among these key stakeholders were additionally discussed at the Pellston Workshop.

## SCENARIOS THAT CAN INCORPORATE AOPS IN CHEMICAL DECISION MAKING

**Figure 2:**
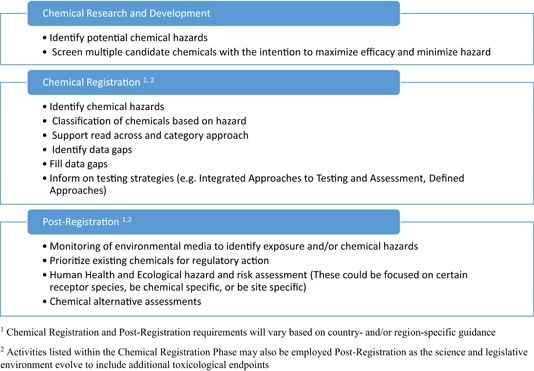
Stages of chemical management within both the regulated and regulatory community with some specific activities listed where adverse outcome pathway frameworks could be applied.

The AOP framework can be applied to a variety of chemical decision‐making scenarios that take place over various stages of chemical management (e.g., research and development, chemical registration for manufacture and sale, and postregistration activities) (Figure 2). Although the same decision (e.g., hazard identification) may be relevant to different scenarios, the considerations may vary from the perspective of the chemical developer or regulator, and may also vary based upon legislation for different jurisdictions or chemical sectors. Chemical companies may define measures to support development of new chemicals (e.g., cost, effectiveness, efficiency, specificity, predicted use, hazard profile), whereas regulatory decisions affect a broader range of stakeholders and are generally determined by statutory requirements that vary based on the chemical sector (e.g., pesticides, industrial chemicals) and/or the potential exposure (sometimes reflected by the volume of production). Despite the different drivers for stakeholders and different requirements for demonstrating chemical safety, AOPs can be useful for a variety of scenarios.

## FIT FOR PURPOSE CONSIDERATIONS

Previous reviews of AOP utility for regulatory decision making have recognized that determining whether an AOP is fit for purpose varies with context (i.e., for routine processes such as reregistration of chemicals already in the market vs emergency situations for chemical spills); however, there has been little discussion of possible criteria for making this determination (Edwards et al. 2016; Kleinstreuer et al. 2016). Judging fit for purpose includes consideration of the AOP and associated assays used to measure KEs within the AOP (Patlewicz et al. 2015). Suggested criteria for judging whether AOPs and associated assays are fit for purpose are listed in Table 1.

**Table 1:** Proposed criteria to assess whether an Adverse Outcome Pathway (AOP) and associated bioassays are fit for purpose

Criteria	Description of criteria	Categories in each criterion
Types of AOPs	The criteria used to describe the types of AOPs relate to the extent of data surrounding KEs and key event relationships KERs. The Putative, Qualitative, Quantitative categories are adapted from Villeneuve et al. (2014).	Putative AOP: Assembly of a hypothesized set of KEs and KERs supported primarily through biological plausibility and/or statistical inference. Assembly of a partial AOP with incomplete linkages between the MIE and AO as a result of known data gaps and uncertainties. Qualitative AOP: Assembly of KEs supported by descriptions of how the KEs can be measured and KERs supported by empirical evidence in addition to plausibility or statistical inference, along with qualitative evaluation of the overall WoE supporting the AOP. A qualitative AOP does not contain threshold information for transitioning from one KE to another. Quantitative AOP: Assembly of KEs supported by mathematical descriptions of the KERs and the accuracy and precision with which the measurements are made, supported by quantitative understanding of what magnitude and/or duration of change in the upstream (preceding) KE is needed to evoke some magnitude of change in the downstream (subsequent) KE. A quantitative AOP contains biological thresholds for KER, or KE weighted in terms of AO probability (OECD 2016a).
Level of complexity of AOPs	Although AOPs may be interconnected across biological systems, they are first developed as more simple, linear constructs. Over time, connections to other AOPs at shared KE nodes may be developed and/or elucidated, such that a network of related AOP emerges (Knapen et al. 2018).	Linear AOP: Exists as a progression from MIE through consequent KEs to an AO without interactions from other AOPs at shared KE nodes. Network AOP: An assembly of 2 or more AOPs that share one or more KEs.
Confidence in the AOP	The level of confidence in the overall AOP is assessed by a WoE approach considering the KEs and KERs and their associated biological plausibility, essentiality, and empirical evidence (in that weight order) as described by Becker et al. (2015) and OECD (2016a, 2016b).	Low confidence: A WoE assessment of the AOP indicates that there is overall low confidence in the AOP in consideration of biological plausibility, essentiality, and empirical evidence. Moderate confidence: A WoE assessment of the AOP indicates that there is overall moderate confidence in the AOP in consideration of biological plausibility, essentiality, and empirical evidence. High confidence: A WoE assessment of the AOP indicates that there is overall strong confidence in the AOP in consideration of biological plausibility, essentiality, and empirical evidence.
Level of external review of the AOP	The AOP status in the AOP‐Wiki refers to the review process established by the OECD and included in the AOP knowledgebase (OECD 2016a, 2016b, 2016c, 2016d; AOPWiki 2019a). For reference, see https://aopwiki.org/oecd_page	Under development: o Open for citation and comment, or o Under development; not open for comment; do not cite. Under review by the EAGMST: o Open for citation and comment, or o Open for comment; do not cite. Approved by the EAGMST. Endorsed by the WNT and the WPHA.
KE in the AOP is of regulatory interest	The AOP includes a MIE, KE, or AO that is used for regulatory decision making. There is either an unambiguous connection to a regulatory endpoint (e.g., skin or eye irritation, sensitization, genotoxicity or mutagenicity, parameters linked to reproductive and/or developmental toxicity, endocrine‐disrupting properties, population‐level adverse effects in environmental organisms) or the AOP is several steps removed from an endpoint of regulatory interest.	Ambiguous connection: There is a more tenuous connection with the measurement and/or assessment endpoint of regulatory interest. Unambiguous connection: There is a clear, empirical connection to the measurement parameters and an assessment endpoint of regulatory interest.
Assay methods	Bioassay methods for investigating KEs in AOPs may be extensively standardized at the international or national level or may have been more recently developed without undergoing formal validation. Sufficient confidence in assay methods may be achieved through use of well‐described methods and assay performance in the context for which it is to be applied (ICCVAM 2018).	Nonvalidated and not well‐described methods Nonvalidated but well‐described methods (e.g., according to OECD GD 211 [OECD 2014b]) Nationally validated: The methods for chemical safety regulation have undergone national validation (e.g., USEPA) Internationally validated: The methods for chemical safety regulation (e.g., OECD Test Guideline) have undergone international validation or meet explicit performance criteria.

AO = adverse outcome; AOP = adverse outcome pathway; EAGMST = Extended Advisory Group on Molecular Screening and Toxicogenomics; GD = guidance document; KE = key event; KER = key event relationship; MIE = molecular initiating event; OECD = Organisation for Economic Co‐operation and Development; USEPA = United States Environmental Protection Agency; WNT = Working Group of the National Coordinators of the Test Guideline Program; WoE = weight of evidence; WPHA = Working Party for Hazard Assessment.

One consideration for judging fit for purpose is the type of AOP based on the level of data supporting the key event relationships (KERs). The level and type of data supporting KERs progressively increases, going from putative to qualitative and from qualitative to quantitative AOPs (Villeneuve et al. 2014; Table 1). The type of AOP—whether putative, qualitative, or quantitative—is not characterized by clearly defined boundaries, but is a general reflection of the level of supporting information that exists on a continuum, and designation into one of these categories often requires expert judgment. Several guiding principles for designating the type of AOP have been provided (Villeneuve et al. 2014). Putative AOPs are characterized by hypothesized and plausible KEs and KERs with incomplete linkages and known data gaps. Qualitative AOPs have KERs that are supported by empirical data, but these KERs do not contain threshold information for consistently transitioning from one KE to another. A quantitative AOP contains measurable KEs with mathematically characterized KERs predictive of the magnitude and/or duration of change in sequential KEs and are predictive of dose–response or time‐course relationships between MIE and AO (Villeneuve et al. 2014).

Adverse outcome pathways are tools for simplifying and characterizing extraordinarily complex biology, and initial development of an AOP construct generally begins with a linear model sorted by level of biological organization (e.g., molecular, cellular, tissue level). As the biology is better understood, the AOP can be further elaborated to better represent biological feedback loops and intersections with other pathways. Fully populated, linear AOPs exist as a progression from MIE through consequent KEs to an AO, whereas network AOPs are an assembly of 2 or more AOPs that share 1 or more KEs (Knapen et al. 2018; Table 1).

The confidence (i.e., high, medium, or low) in a particular AOP construct is assessed by a weight‐of‐evidence (WoE) approach that considers the KEs and KERs in terms of their biological plausibility, essentiality, and empirical evidence (Becker et al. 2015; OECD 2016a, 2016b; Table 1). Biological plausibility refers to the level of biological knowledge in support of the mechanistic linkages underpinning KERs in the AOP. Essentiality refers to the level of evidence available indicating that “upstream” KEs in the AOP are pivotal or essential for subsequent KEs to occur. The empirical evidence refers to the level of dose–response, temporal, and incidence concordance data in support of the AOP (Becker et al. 2015).

To establish scientific confidence in the AOP and its components, a review process has been established (OECD 2016b, 2017a) and is indicated in the AOP‐Wiki (AOPWiki 2019a) (https://aopwiki.org/oecd_page), which serves as the primary international repository for AOP development efforts (OECD 2016a, 2017a; Table 1). AOP confidence information may be provided in the AOP‐Wiki by developers (i.e., in the “Essentiality of the Key Events” section or in the “Evidence Assessment” section). If this information is not present in the AOP‐Wiki (which may be the case for AOPs still in development), the confidence level may be independently assessed by AOP practitioners according to the principles described by Becker et al. (2015) and the Organisation for Economic Co‐operation and Development (OECD 2016a, 2016b).

The Society for the Advancement of Adverse Outcome Pathways (https://www.saaop.org/) responds to changes needed in the AOP‐Wiki to ensure consistency with the OECD AOP guidance and handbook documents and to foster a crowd‐sourced approach to AOP development (OECD 2016c). The OECD's AOP development program is overseen by the Extended Advisory Group on Molecular Screening and Toxicogenomics (EAGMST; http://www.oecd.org/chemicalsafety/testing/adverse‐outcome‐pathways‐molecular‐screening‐and‐toxicogenomics.htm), members of which are nominated by OECD member country National Coordinators, to assess an AOP's adherence to guidance principles, completeness, and scientific robustness. After EAGMST approval, the Working Group of the National Coordinators for Test Guidelines (WNT) and the Working Party on Hazard Assessment (WPHA) are responsible for endorsement of AOPs (OECD 2017a). In general, most AOPs are expected to contain at least 1 KE that is considered adverse (i.e., the AO) from a regulatory decision‐making perspective. However, there are some instances in which AOP development may outpace or diverge from regulatory perceptions of adversity; thus an assessment of the AOP for KEs that are relevant in a regulatory context is important for consideration (Table 1).

Although characteristics of the individual AOP construct are important to consider for judging fit for purpose, the level of information and confidence in assays that are used to measure KEs and KERs in an AOP are also important to consider. Assays may have gone through validation either at the national or international level and been certified in ring‐trials between labs to demonstrate relevance, reliability, and transferability of methods (OECD 2005), while other assays may not have undergone ring‐testing but are peer reviewed, well described, transferable, and follow principles designed to provide confidence in the resultant data (OECD 2014b). In other cases, methods may not be as thoroughly vetted according to defined validation principles. Fit‐for‐purpose considerations should involve regulators and the regulated community, and may not necessarily require formal, traditional ring‐trial validation exercises to have appropriate confidence associated for application to regulatory contexts (ICCVAM 2018). Taken together, the characteristics of both the AOP and the associated assays should be considered when evaluating whether they are fit for purpose in different chemical management scenarios (Table 1).

Illustrated in the next section are several examples of chemical decision‐making scenarios, with an examination of how the AOP framework has been or could be applied in these scenarios; in each case, criteria listed in Table 1 are considered. The intention here is to present case studies to illustrate the application of AOPs and fit‐for‐purpose considerations in different contexts, which can inform the use of AOPs in applied contexts in the future (Carusi et al. 2018).

## EXAMPLE SCENARIOS IN WHICH AOPS CAN INFORM CHEMICAL DECISION MAKING

### Bioassay identification and development

Researchers and chemical managers can use the AOP framework to identify KEs that inform assay selection and/or future assay development for a variety of scenarios related to chemical decision making. An important step to building capability to assess potential hazards of chemicals is the identification of bioassays, whether in vitro or in vivo, that are indicative of potential adverse effects and are cost effective and relatively straightforward to conduct in a laboratory setting. It may be desirable to use assays for KEs that are simple to measure, can be causally linked to an AO, or are a point of convergence in an AOP network (Villeneuve et al. 2018). For example, although incomplete in some cases, identification and development of assays based on KEs have already been developed and applied in the following chemical decision‐making scenarios: 1) early identification of potential hazards during chemical research and development (e.g., drug development) (Carney and Settivari 2013), 2) prioritization of existing chemicals for further regulatory activities (Browne et al. 2017), and 3) environmental monitoring (Neale et al. 2017) (Figure 2). These scenarios span different stages of chemical management activities (i.e., early research and development, postregistration; Figure 2), and the strategic identification and development of assays based on KEs which potentially lead to an adverse response can contribute to chemical management decisions in these different scenarios.

#### Assay identification and development for hazard assessment during chemical research and development

The AOPs can be used to identify particularly pivotal, informative, and/or easy to measure KEs that are the basis for screening assays employed in the earliest stages of research and development. Industry pioneered the development and use of in vitro and in silico tools to evaluate the potency, specificity, and efficacy of chemicals during early development. The advantage to developing AOPs around existing assays and developing assays around AOPs is that predictive screening tools can be linked to endpoints that are a concern from a regulatory and public perception perspective (e.g., genotoxicity, endocrine activity, irritation or corrosion, and sensitization). For example, developmental neurotoxicity is an endpoint of concern from both a regulatory and a public perception perspective, and this adverse effect can occur through a variety of molecular initiating events. One particular approach to screening for developmental neurotoxic effects is to measure alterations in firing rate among in neuronal networks grown in vitro on microelectrode arrays (Valdivia et al. 2014). This type of in vitro bioassay has the capacity to integrate effects from a variety of different initiating events, and would therefore be a promising bioassay to develop for screening potential neurotoxicants (Valdivia et al. 2014).

**Figure 3:**
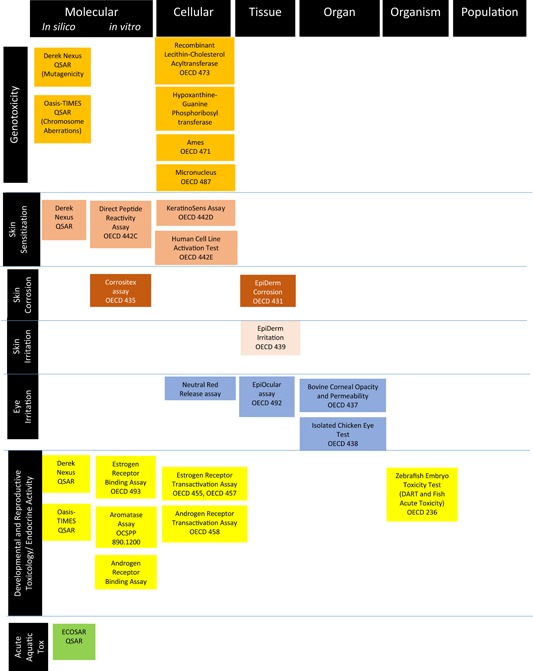
Example early screening battery for industrial chemicals (later stage testing not specified). It should be noted that screening with hazard‐based assays alone is not adequate for assessment in early product development phases. Predictions of environmental and human exposure, environmental fate (e.g., bioaccumulation and persistence), and toxicokinetics should also be included in early screening to ensure a risk‐based approach to chemical decision making. Generally assays would be deployed in a tiered manner based on outcomes from earlier tiers. Note: Various test guidelines under OECD and OCSPP of the EPA are included where available. DART = developmental and reproductive toxicology; ECOSAR = ecological structure–activity relationship; OCSPP = office of chemical safety and pollution prevention; OECD = Organisation for Economic Co‐operation and Development.

The average cost of developing a new pesticide is estimated to be US$286 million (Sparks and Lorsbach 2017). The cost of a new prescription drug is estimated to be $2.9 billion (DiMasi et al. 2016), and a recent analysis indicated more than 30% of drugs are withdrawn from the market due to deleterious effects (Downing et al. 2017), including several high‐profile cases that resulted in lawsuits. Well‐developed AOP networks that are leveraged to develop informative assays can guide selection of safer candidate compounds and avoid such postmarketing issues, resulting in considerable cost savings. Figure 3 provides an example of a toxicity screening battery that consists of in silico and in vitro assays for evaluating the safety of candidate chemicals during research and development. These predictive tools can be deployed in a tiered process, with results from earlier tests informing the need for subsequent testing at higher levels of biological organization. If warranted, in vitro studies can be confirmed using targeted in vivo tests.

For assay identification and/or development of assays deployed during early screening efforts within the commercial sector, AOPs at nearly any level of development or complexity (i.e., putative, qualitative, quantitative, linear, network) can be useful. Well‐described, high‐confidence, externally reviewed or endorsed AOPs will increase the confidence associated with using AOPs to predict potential toxicity or activity. However, for applications in early chemical screening, even putative, low confidence, partially characterized AOPs that have not undergone extensive independent review can be helpful for bridging data gaps and predicting chemical effects—especially for new chemicals when very little information may be available although confirmation may be needed. Chemical screenings during early research and development are conducted for a variety of reasons, including avoidance or minimization of hazard, or relative comparison of 1 chemical structure to another using an endpoint of interest. Thus, the endpoints under investigation during early screening efforts may or may not be of direct relevance to chemical regulatory issues. For chemical companies, validation of assays used during early screening efforts is essential for evaluating chemical safety, but confidence may be established based on internal testing of the reliability of the assays. Many large chemical companies develop their own predictive tools for early screening‐level decisions, and there is no requirement for external validation. However, as evidenced by the example screening battery indicated in Figure 3, many commonly employed assays in company screening efforts have already been formally validated at the national and/or international level. Practically speaking, assays that have already been validated by reputable agencies (e.g., OECD, US Environmental Protection Agency [USEPA], American Society for Testing and Materials [ASTM]) allow the chemical industry to leverage these vetted assays without having to develop new assay tools “from scratch” based solely on internal efforts.

#### Assay identification and development for use in prioritization of existing chemicals for regulatory action

Adverse outcome pathways can guide assay identification and development that is then leveraged to prioritize chemicals for potential regulatory activities in the postregistration stage of chemicals management (Figure 2). With the estimated 80 000 existing chemicals and new chemicals coming onto the market annually (IOM 2014), regulatory efforts to evaluate chemical hazards are strained based on the sheer number of chemical compounds for consideration. Therefore, methods are needed for rapidly identifying the chemicals that are most likely to be hazardous and prioritizing those for further regulatory assessment and management. The USEPA (2019a) Endocrine Disruptor Screening Program (EDSP) provides an example of using AOPs to target assay development in order to link a MIE to an AO of regulatory concern with respect to the estrogen, androgen or thyroid signaling pathways.

The EDSP Tier 1 guideline tests (USEPA 2019b) include several in vivo assays to measure chemical interactions with the thyroid hormone pathway but, to date, do not include any mechanistic assays for thyroid targets, and therefore, it may be difficult to link an apical in vivo response to a thyroid mechanism of action (Browne et al. 2017). In addition, relying exclusively on animal testing to screen chemicals for potential endocrine disruption limits the number of chemicals that can be screened. In an effort to remedy this gap in mechanistic assays, the USEPA has proposed a thyroid AOP network to guide bioassay development and selection based on defined KEs (OECD 2014a; USEPA 2017). The USEPA identified 15 potential MIEs for thyroid‐based AOPs, including those related to the biological processes of thyroid hormone synthesis, transport, binding to nuclear receptors, and effects in peripheral tissues (USEPA 2017; Villeneuve et al. 2018). The USEPA then prioritized the identified MIEs based on their relevance to the thyroid pathway, their toxicological potential, and current status of high‐throughput (HTP) bioassay development. Four MIEs from this thyroid AOP network, including the sodium iodide symporter, thyroperoxidase, iodothyronine deiodinase, and hepatic nuclear receptors involved in thyroid metabolism, were ranked as high priority for bioassay development for chemical screening and prioritization in the EDSP (USEPA 2017). Recent bioassay development has been undertaken to measure sodium‐iodide symporter and the iodothyronine deiodinase MIEs and other high‐priority targets (USEPA 2017; Hornung et al. 2018; Wang et al. 2018).

In this example, several MIEs and early KEs that are potential targets for chemicals that affect the thyroid hormone pathway have been identified (OECD 2014a; USEPA 2017), although the full AOP networks linking these events to AOs may not be well defined. Based on the multiplicity of events that are known to interfere with the thyroid axis, a network AOP is best suited to characterize the various interactions that can affect the vertebrate thyroid pathway. Because the intent is to identify and/or develop bioassays for eventual use in regulatory efforts to prioritize additional chemical assessments, the confidence in the AOP network should be moderate or high. In this case, it is not critical for the thyroid network AOP to undergo formal external review prior to use in a regulatory context; however, as additional information is added to the AOP‐Wiki, individual AOPs in this network should be a high priority for external review, which would increase the overall confidence in the AOP network. Because interferences with the thyroid pathway have already been specifically identified by the USEPA's EDSP as an area of concern, the KEs in the thyroid pathway network are already established as having high regulatory interest (USEPA 2017). Ultimately, these assays will be applied in regulatory prioritization activities, and therefore, should contain well‐described methods (e.g., according to OECD GD 211 on describing nonguideline in vitro test method; OECD 2014b) or be more formally validated, either nationally or internationally.

#### Assay identification and development for use in environmental monitoring activities

Assays based on KEs from AOPs are also being identified and developed for environmental monitoring purposes (Schroeder et al. 2016). For example, the SOLUTIONS project (Helmholtz Centre for Environmental Research 2019) in the European Union focuses on assembling a battery of assays for water quality monitoring and chemical assessments (Neale et al. 2017). Molecular initiating events or early KEs that are most useful as the basis for bioassay development for use in environmental monitoring are those with broad coverage of the biological effects elicited by chemical contaminants (e.g., endocrine disruption, oxidative stress responses, cytotoxicity), are linked to AOs relevant to environmental health, and pertain to effects that have previously been observed in the environment (Escher et al. 2014; Neale et al. 2017). The AOP framework can be used to identify a battery of new or existing assays that measure the biological effects associated with environmental chemicals exposures.

Bioassays based on putative AOPs could be useful for environmental monitoring efforts, given that the focus is generally on screening for biological activity. Although there is no strict requirement for an AOP or AOP network to be in existence to develop a bioassay for use in environmental monitoring, when those AOPs are present, they provide an interpretive context that can make the plausible connections between chemical exposure and biological hazards more apparent. Additionally, if available, consideration of results in an AOP network context can provide insights into effects of chemical mixtures and multiple coincident stressors. If the intent of monitoring is hazard determination for a specific chemical, then greater confidence in the AOP (i.e., moderate or high) is needed. In general, an “Endorsed” or “Approved” status in the AOP‐Wiki (Table 1) will add confidence to the use of the AOP for bioassay development in environmental monitoring programs; however, a strict requirement for this level of external review is not needed in all scenarios. An AOP that is “Under Development” or “Under Review” can still supply sufficient information for bioassay development for use in environmental monitoring. Most environmental monitoring efforts are focused on identifying chemical presence or biologic activity; therefore, the connection to a regulatory endpoint may not be required, although an understanding of such would place the quantity measured in an environmentally relevant context. Because assays are generally leveraged in environmental monitoring programs in a screening‐level capacity to identify areas of concern for further investigation, external validation of the assay methods at the national or international level is not strictly necessary. However, if environmental monitoring assays are used to guide a regulatory action, then the validation status of the assay should adhere to a higher standard.

### Adverse outcome pathways inform testing strategies such as defined approaches

There is global interest in reducing, refining, and replacing the use of animals in chemical safety testing (Flecknell 2002), and in several regions and chemical sectors, there are regulatory requirements to find alternatives to animal testing (e.g., European Union 2003; USEPA 2016a). Even in jurisdictions where animal testing is permitted, there is interest in evaluating all available data to determine whether additional testing is needed and developing “intelligent testing strategies” (Van Leeuwen et al. 2007). To aid in the organization of existing, often disparate data, guidance has been developed for using Integrated Approaches to Testing and Assessment (IATA) which can be informed by AOPs (OECD 2016a). The IATAs can be used to integrate and weigh relevant existing data in a WoE assessment, to inform decision making regarding potential chemical hazard and/or risk, and to guide the generation of new data (OECD 2016a). Adverse outcome pathways have informed IATAs in regulatory decision making for several acute toxicity endpoints including skin irritation, skin corrosion, eye irritation, and skin sensitization (OECD 2014c, 2016a, 2017b; Tollefsen et al. 2014). Integrated Approaches to Testing and Assessment are designed to meet specific regulatory needs and are intended to be flexible approaches for gathering available information and organizing data, and WoE assessments can be determined by expert judgment. Some elements within an IATA can be standardized. For example, similar to harmonized test guidelines, data can be generated using standardized information sources (e.g., in vitro assays or in silico methods) and resulting data can be evaluated using fixed data interpretation procedures (OECD 2016a, 2016c). Standardizing the information sources and data interpretation procedure for integrating the resulting data removes the expert judgment and is called a “defined approach” (OECD 2016d).

Adverse outcome pathways can be highly valuable as an organizing construct for developing defined approaches for use in regulatory decision making. Defined approaches represent the increased understanding of biology leading to AOs, coupled with the improved alternative methods for predicting in vivo responses. Although every assay has inherent strengths and limitations, defined approaches leverage the strength of more than 1 method and thereby may reduce assay limitations when methods are used in combination. These standardized approaches for integrated testing are likely to be increasingly common and can be constructed with a regulatory endpoint in mind (e.g., endocrine disruption, skin sensitization, lethality). Adverse outcome pathways can also support the development of defined approaches that use validated methods and are amenable to harmonization and regulatory uptake.

Several examples of defined approaches for skin sensitization were recently detailed in Annex 1 of an OECD guidance document (OECD 2016d). In this case, the complete linear AOP for skin sensitization has been well described and endorsed (AOPWiki 2019b), and there are multiple internationally validated test guidelines to measure all KEs along the AOP (http://www.oecd‐ilibrary.org/environment/oecd‐guidelines‐for‐the‐testing‐of‐chemicals‐section‐4‐health‐effects_20745788). The level of confidence in the AOP underlying the biological sequelae from MIE to AO and the availability of validated assays to measure KEs in the pathway has led to the USEPA's acceptance of defined approaches in lieu of animal data to satisfy the regulatory requirement for skin sensitization testing (USEPA 2019c).

### Adverse outcome pathways inform ecological species management

**Figure 4:**
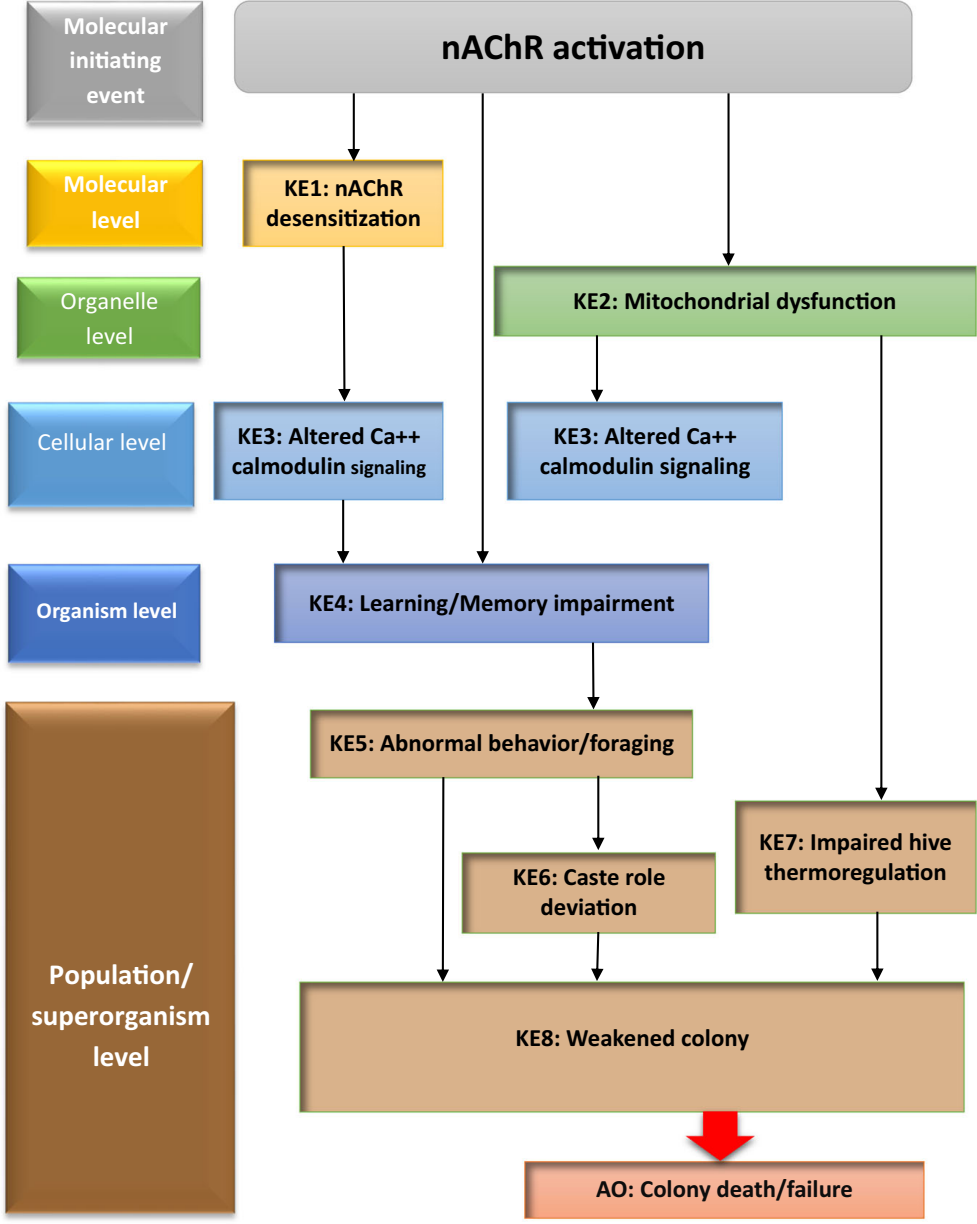
Putative honeybee (*Apis mellifera*) nAChR AOP depicting the MIE and subsequent KEs leading to an AO. AO = adverse outcome; AOP = adverse outcome pathway; KE = key event; MIE = molecular initiating event; nAChR = nicotinic acetylcholine receptor.

Adverse outcome pathways were originally conceived as a framework to aid ecotoxicology research and risk assessments (Ankley et al. 2010). Appropriately, AOP constructs have recently been used to help understand the decline of honeybees (*Apis mellifera*). Honeybees play a critical role as pollinators in many ecosystems (Potts et al. 2010), and based on particular concerns for the health of honeybee colonies, one recent AOP network was constructed to organize information concerning chemical and nonchemical perturbations leading to honeybee colony failure (LaLone, Villaneuve et al. 2017; AOP numbers 77–82 and numbers 84–90 at https://aopwiki.org/aops; Figure 4), and to better understand potential factors affecting honeybee colony health. Thirteen AOPs in the honeybee AOP network begin with the MIE nicotinic acetylcholine receptor (nAChR) activation, with subsequent KEs leading to an AO of weakened colony performance and eventual colony failure (Figure 4; LaLone, Villaneuve et al. 2017). Although chemical stressors may contribute to declines in honeybee colonies, numerous other stressors are thought to influence honeybee colony health, including but not limited to pathogens and parasites, loss of habitat, and climate change (Potts et al. 2010).

The nAChR AOPs for colony failure are described as putative, and the WoEs for the KERs vary from weak to strong (LaLone, Villaneuve et al. 2017). If the AOP were to be applied to predict adverse effects in an ecological risk assessment scenario, then the KERs used to predict the AO would need to have a higher level of confidence. Currently, the 13 individual AOPs that represent part of the network of effects of nAChR on honeybee colony failure have the status “Under Development” in the AOP‐Wiki, but even so, these AOPs are adequate for uses related to gaining greater insight into factors affecting honeybee colonies (AOP numbers 77–82 and numbers 84–90; https://aopwiki.org/aops), and this understanding contributed to 2017 USEPA policies to mitigate risk to bees (USEPA 2019d). Honeybee colony failure is of interest to the public and to regulators, and thus, this AO has an unambiguous connection to an assessment of regulatory interest (i.e., impaired survival). Although the current status of the AOP network has improved the understanding of the multifactorial process of colony failure and directed research efforts to bridge knowledge gaps, using the network AOP to quantitatively predict adverse effects in an ecological risk assessment scenario would require development and validation of additional assays measuring KEs, and the AOP network would require additional characterization to predict the AO with a higher level of confidence. Further, the decline of honeybees is a global issue, and OECD review, approval, and endorsement of the AOPs would increase confidence of the network AOP for informing international regulatory decisions.

### Adverse outcome pathways inform endpoint selection in ecological risk assessments

The requirements for demonstrating “adversity” can differ between evaluation of chemical effects relevant to human health (i.e., effects in an individual) and ecotoxicology (i.e., effects in populations). It is often challenging to link the effects of a chemical to population‐level responses, given that empirical data directly assessing population effects are relatively rare, and the health of a population is influenced by a host of factors in addition to chemical contaminants (e.g., species competition, disease, pests, habitat quality, weather events). However, AOPs can be used to address these challenges by organizing available data based on increasing level of biological complexity and increasing relevance to population‐level AOs. This organizational structure inherent in ecological AOPs can assist in study design and endpoint selection in ecological risk assessments.

For example, various AOPs exist for compounds that bind to the aryl hydrocarbon receptor (AhR) and induce adverse effects in wildlife (e.g., dioxins, furans, and coplanar polychlorinated biphenyls; Van den Berg et al. 1998). Specifically, an AOP based on AhR activation as an MIE and leading to alterations in cardiovascular development resulting in early life stage mortality in teleost fish and birds has been recently developed and is currently undergoing revisions by the OECD (see AOP number 21 at https://aopwiki.org/aops). The AOP‐predicted effects in birds also include such things as pericardial edema, developmental abnormalities and embryo mortality leading to population decline (see AOP numbers 150 and 131 at https://aopwiki.org/aops). Available information provided in these AOPs can help ecological risk assessors select assessment and measurement endpoints for ecological risk assessments of dioxin‐like chemicals in local bird populations. By leveraging the information available in these AOPs, risk assessors would be advised to include, for example, measurement endpoints related to bird developmental abnormalities and embryo mortality in an ecological risk assessment for dioxin‐like compounds. Additionally, because of the differential sensitivity across avian species due to amino acid variability in the ligand binding domain of the aryl hydrocarbon receptor (Farmahin et al. 2013), particularly sensitive bird species may be selected for inclusion in an ecological risk assessment with dioxin‐like contaminants, contingent also upon their habitat range and suitability as an ecological monitor.

In this ecological risk assessment scenario, the AOP framework is primarily applied to inform study design, and the fit‐for‐purpose criteria listed in Table 1 are considered accordingly. Using AhR activation as an MIE is well supported by data and there are defined causal linkages leading to a population‐relevant AO, thereby providing adequate information to use the AOP to make informed decisions about an ecological risk assessment study design. Thus, this example demonstrates that a fully populated AOP with detailed KERs for each node along the pathway is not necessary for all decision‐making scenarios. In this specific example, the well‐characterized MIE contributes to the evaluation of species sensitivity differences, and there is a well‐understood relationship from the MIE to the AO. Because the AOP is used in this case to inform study design, it is also not necessary that it be quantitative in nature. There should be at least a moderate level of confidence in the AOP, and an “endorsed” or “approved” status of the AOP could be advantageous, but the absence of this status does not affect the fact that the AOP is supported by data indicating biologically plausible relationships between KEs. The AO should have an unambiguous linkage to endpoints of regulatory interest for ecological risk assessment (i.e., endpoints that are informative for predicting population‐level metrics). If AhR activation is measured to determine the potential MIE to help inform the study design for a site‐specific ecological risk assessment of dioxin‐like chemicals, then such assays would not necessarily require external validation, but should be well described to ensure that the results are reliable.

### Adverse outcome pathways can support decision making in emergency spill scenarios

In some cases, unforeseen circumstances or chemical accidents preclude the development of prospective studies and regulators are forced to make immediate decisions with available and readily accessible information. The 2010 Deepwater Horizon oil spill released an estimated 2.94 million gallons of oil into the Gulf of Mexico and was the largest marine oil spill in United States history. In efforts to protect marine and estuarine ecosystems, more than 350 000 gallons of oil dispersants were used to disrupt rafts of surface oil (USEPA 2016b); however, limited data were available regarding their potential toxicity. The USEPA Office of Research and Development evaluated 8 commercially available oil dispersants to inform selection and mitigate hazards for aquatic wildlife. Selected HTP in vitro assays were used to screen for endocrine activity, interaction with hepatic nuclear receptors for xenobiotic metabolism, and cytotoxicity (Judson et al. 2010). These assays were selected because they were part of well‐characterized, putative AOPs (https://aopwiki.org/) that are conserved across vertebrate taxa. The primary oil dispersant selected to dispel the oil rafts did not have detectable estrogen receptor activity in the HTP assays (Judson et al. 2010). Thus, potential MIEs and early key events in well‐characterized pathways can be used to mitigate probable ecological risks and make time‐sensitive decisions.

In this particular scenario, despite the fact that AOPs were not fully described, chemical interactions with the estrogen receptor and androgen receptor are MIEs for a variety of putative AOPs (Browne et al. 2017). This example can serve as a reminder that the AOP framework and supporting assays can be applied in emergency situations despite associated uncertainties, lack of complete AOP description or external review, or use of validated test methods to measure events within the AOP. Emergency scenarios in which an assessment is needed in a short timeframe are examples of situations where AOPs can be applied quickly by decision makers. Thus, this example emphasizes the value in proactively and collaboratively developing AOPs and assays as well as storing information in a database that can be accessed quickly for available AOP knowledge.

## DISCUSSION

### Fit‐for‐purpose considerations

Certain themes are apparent when the use of AOPs and associated assays are evaluated in chemical decision‐making scenarios using our proposed fit‐for‐purpose criteria (Table 1). Clearly, context plays a critical role in determining what is fit for purpose. For example, generally AOPs and associated assays used to inform regulatory decisions needed higher confidence levels than those for nonregulatory decision‐making efforts. Another theme is that when emergency situations are encountered, ultimately some information that assists in rapid decision making is better than none, despite potential data gaps or the level of uncertainty.

Obviously, a moderate to high level of confidence is preferred for any tool applied to decision making. For decision contexts that have fewer consequences (such as screening‐level assessments for chemical selection purposes), AOPs with limited supporting evidence and, therefore, low confidence may nonetheless be useful. Another theme is that AOP may not be fully characterized or may have low confidence in KEs or KERs, but if the portion of the AOP that links a measured KE to the AO has moderate or high confidence, this information can be very informative for chemical decision making. It is recognized that AOPs are pragmatic representations of biological processes, and AOP networks are generally intended to be representative of more complex biological and environmental scenarios. Adverse outcome pathway networks better recapitulate realistic interactions, and although fully described networks are not a requirement for use, both the understanding of events and the predicting of outcomes are improved by incorporating AOP networks. If filters and layers are integrated in the AOP‐Wiki as described by Knapen et al. (2018), the utility of AOP networks for regulatory purposes will likely increase due to the enhanced ability to tailor AOP networks to suit particular research questions or applications. The AOP review status in the AOP‐Wiki can lend confidence for applying AOPs to chemical decision making; however, because this process is not necessarily tied to the level of detail or confidence in the AOP based on WoE evaluation of KERs or the relevance and reliability of associated assay tools, a strict requirement of external review of the AOP in the AOP‐Wiki is not needed for many scenarios. However, if the AOP is highly leveraged in regulatory decision making (e.g., used to develop defined approaches applied in regulatory assessment schemes), then the process of review and endorsement of the associated AOPs should be considered.

It must also be noted that AOP development and interpretations vary with respect to human health versus ecotoxicology. For human health decision making, effects on individuals can be considered adverse. In addition, impaired individual survival, growth, development, or reproduction is clearly unacceptable for individuals exposed to chemicals, and thus, regulatory decisions are based on changes to KEs upstream of the AO and with considerable safety factors built in to account for variability in sensitivity. In contrast, population‐level changes are considered AO for ecotoxicology and these are not only difficult to directly measure, but apical responses used in regulatory decision making are very general health outcomes and can be influenced by a multitude of biotic and abiotic pathways following exposure to chemicals or a variety of nonchemical stressors. Further, the laboratory model species used to measure biological responses following chemical exposure are generally selected for their reliable growth, development, and reproduction; however, these species may be particularly poor sources of information upon which to build ecotoxicology AOPs in terms of their potential range of sensitivities.

The AOP framework is relatively new and continuing to evolve. The AOP Knowledge Base (AOP‐KB; OECD 2019) provides the most complete and comprehensive resource for AOP developers and users, but to some extent, the information populating the AOPKB to date has been essentially proof‐of‐concept. The specific AOPs included were determined by the research interests and dedicated efforts of the voluntary developers. To improve regulatory uptake, AOPs should be designed and developed to satisfy a regulatory need. In this context, the AOP development would begin with the regulatory decision (e.g., screening, prioritization, read‐across, predictive model building, environmental risk assessment), include regulatory endpoints as key events, and have well‐developed tools (e.g., internationally adopted or at least well described and with known performance assays) to measure these events. Furthermore, a proper justification of the overall methodology to ensure sufficient confidence in results is needed to improve regulatory uptake of AOPs (ICCVAM 2018). In other words, AOPs intended to be used in a regulatory context should be built for that purpose.

### Further recommendations to enhance application of AOPs in chemical management decisions

Based on discussion at the SETAC Pellston Workshop on “Advancing the Adverse Outcome Pathway Concept: An International Horizon Scanning Approach,” our workgroup discussed ways to further enhance the use of AOPs to inform and support chemical decision making. The following is a summation of those discussions as a series of recommendations:

- The elements for consideration in terms of the fit for purpose outlined in Table 1 could be further refined to provide a basis for practitioners to assess the utility of AOPs in specific contexts. We have provided an initial list of potential fit‐for‐purpose considerations; however, these may be refined or additional criteria may be added as more examples of AOPs and associated assays are applied to chemical decision making.
- Priority should also be given to developing AOPs that are relevant to a large number of chemicals and associated modes of action. Pathways that include KEs, such as oxidative stress or membrane disruption, which may be affected by many chemicals should be prioritized for future work. These AOPs can then be used to prioritize the backlog of thousands of industrial chemicals for additional testing.
- Priority should also be given to developing AOPs for relatively specific endpoints (e.g., respiratory sensitization) and complex regulatory endpoints (e.g., repeated dose toxicity, reproductive toxicity) in humans, and AOPs with apical effects that impact populations for wildlife species (e.g., impaired population survival, growth, development, and reproduction) where the domain of applicability extends to multiple taxa.
- It is recommended that AOPs and associated assays continue to be applied to the development of defined approaches. Defined approaches, which are built on the mechanistic understanding provided in AOPs, can aid those undertaking chemical assessment to integrate various information in a consistent, systematic and transparent manner.
- There is a need for increased understanding of AOPs among the chemical regulatory community, and an improvement in access points for AOP users. Whereas there may be a basic understanding of the underlying theory of AOPs and an expectation that they can be used in decision making, it is important to also communicate limitations to as well as opportunity for their use or implementation. Informing stakeholder groups can result in increased willingness to address uncertainties that limit the utility of some AOPs and further the development of other AOPs (Carusi et al. 2018).
- It is further recommended that additional tools can be used to increase AOP application in chemical decision making. Specifically, these tools include the following:
  o curated AOPs with articulated level of scientific confidence;
  o increased availability of in silico models and in vitro assays to probe MIEs or KEs and KERs, and a way to increase the visibility of how these approaches are associated with AOPs; and
  o continued development of databases with curated AOP information (AOP‐Wiki, AOP‐KB), with possibilities to filter data. One of the SETAC Pellston workgroups identified potential filters and layers with which to tailor AOPs to address given questions, and several modifications have been proposed to make AOP‐Wiki and AOP‐KB more user friendly (Knapen et al. 2018).
- As noted earlier, the utility of an AOP toward integrating information over multiple levels of biological organization is not limited to fully vetted pathways. To gain regulatory attention though, AOPs and underlying tools must provide a means of ensuring effective hazard characterization, which is achieved through transparency in the process and clarity, consistency, and reasonableness of the hazard assessment product. Transparency is ensured through identification of the methods, key assumptions, and limitations; clarity is ensured through the use of plain language and brevity; consistency is ensured by adherence to established regulatory authority guidelines, policy, or process; and reasonableness is ensured by reliance on high‐quality, scientifically sound data and processes that are consistent with the state of the science.

A concerted effort by all stakeholders on the above elements is needed to continue to advance the use of the wealth of biological knowledge and understanding that are described and communicated in an AOP or AOP network. These advancements will aid with the integration of information from burgeoning numbers of test systems to ensure that human and environmental health and safety of an increasing number of marketed chemicals are effectively, and hopefully more efficiently, addressed.

## Disclaimer

The opinions expressed and arguments employed herein are those of the authors, and do not necessarily reflect the official views of the OECD or of the governments of its member countries, nor the official policy of any federal agency.

## Data Accessibility

There is no associated metadata or calculation tools for this manuscript, which is focused on applied and regulatory science. However, when specifically mentioned, AOPs in the AOP–Wiki are cited with the website.
